# Effectiveness of the 2023–2024 Omicron XBB.1.5-containing mRNA COVID-19 Vaccine (mRNA-1273.815) in Preventing COVID-19–related Hospitalizations and Medical Encounters Among Adults in the United States

**DOI:** 10.1093/ofid/ofae695

**Published:** 2024-11-26

**Authors:** Hagit Kopel, Andre B Araujo, Alina Bogdanov, Ni Zeng, Isabelle Winer, Jessamine P Winer-Jones, Tianyi Lu, Morgan A Marks, Machaon Bonafede, Van Hung Nguyen, David Martin, James A Mansi

**Affiliations:** Medical Affairs, Moderna, Inc., Cambridge, Massachusetts, USA; Medical Affairs, Moderna, Inc., Cambridge, Massachusetts, USA; Real World Evidence, Veradigm, Chicago, Illinois, USA; Real World Evidence, Veradigm, Chicago, Illinois, USA; Real World Evidence, Veradigm, Chicago, Illinois, USA; Real World Evidence, Veradigm, Chicago, Illinois, USA; Medical Affairs, Moderna, Inc., Cambridge, Massachusetts, USA; Medical Affairs, Moderna, Inc., Cambridge, Massachusetts, USA; Real World Evidence, Veradigm, Chicago, Illinois, USA; Epidemiology Consulting, VHN Consulting Inc., Montreal, Quebec, Canada; Medical Affairs, Moderna, Inc., Cambridge, Massachusetts, USA; Medical Affairs, Moderna, Inc., Cambridge, Massachusetts, USA

**Keywords:** 2023–2024 COVID-19 vaccine, hospitalizations, mRNA-1273.815, omicron XBB.1.5 vaccine, vaccine effectiveness

## Abstract

**Background:**

This study aimed to evaluate the vaccine effectiveness (VE) of mRNA-1273.815, a 2023–2024 Omicron XBB.1.5-containing mRNA COVID-19 vaccine, at preventing COVID-19–related hospitalizations and any medically attended COVID-19 in adults.

**Methods:**

In a linked electronic health record–claims dataset, we identified US adults (≥18 years) who received the mRNA-1273.815 vaccine (exposed cohort) between 12 September and 15 December 2023, matched 1:1 to individuals who did not receive a 2023–2024 updated COVID-19 vaccine (unexposed cohort). Cohorts were balanced using inverse probability of treatment weighting on demographics, vaccination and infection history, and underlying medical conditions. Study cohorts were followed until 31 December 2023 for COVID-19–related hospitalizations and medically attended COVID-19. Cox regression was used to estimate hazard ratios and VE. Subgroup analyses were performed for adults ≥50 years, adults ≥65 years, and individuals with underlying medical conditions.

**Results:**

Overall, 859 335 matched pairs of mRNA-1273.815 recipients and unexposed adults were identified. The mean (standard deviation) age was 63 (16) years. More than 60% of individuals in both cohorts had an underlying medical condition. Among the overall adult population, VE was 60.2% (95% confidence interval, 53.4–66.0) against COVID-19–related hospitalization and 33.1% (30.2–35.9) against medically attended COVID-19 over a median follow-up of 63 (interquartile range: 44–78) days. VE estimates by age and underlying medical conditions were similar.

**Conclusions:**

These results demonstrate the significant protection provided by mRNA-1273.815 against COVID-19–related hospitalizations and any medically attended COVID-19 in adults, regardless of vaccination history, and support Centers for Disease Control and Prevention recommendations to stay up-to-date with COVID-19 vaccination to prevent COVID-19–related outcomes, including hospitalizations.

## Teaser

- In US adults (≥18 years), mRNA-1273.815, a 2023–2024 updated COVID-19 vaccine, provided significant protection against COVID-19–related hospitalizations and any medically attended COVID-19.
- Results were consistent among subpopulations of older adults (ages ≥50 and ≥65 years) and those with underlying medical conditions.

COVID-19 continues to be the most common cause of severe outcomes due to viral respiratory illness in the United States and was associated with >900 000 hospitalizations and 75 000 deaths in 2023 [1–3]. In 2023, the average weekly incidence of COVID-19–associated hospitalizations and deaths surpassed the average weekly incidence of influenza and respiratory syncytial virus, according to the US Centers for Disease Control and Prevention (CDC) [1]. The burden of COVID-19 is not limited to the acute phase because even patients with mild disease can develop conditions, including long COVID, that last months following the initial infection [4–6].

Due to the emergence of novel SARS-CoV-2 viral variants and the observed impact on vaccine performance, the US Food and Drug Administration approved and authorized updated formulations of COVID-19 vaccines that were designed to target the circulating Omicron XBB.1.5 variant in the fall of 2023 [7]. Early data suggest that Omicron XBB.1.5-containing vaccines elicit robust and diverse neutralizing antibody responses against XBB.1.5 and other recent circulating variants, including JN.1 [8, 9]. Several studies have examined the vaccine effectiveness (VE) of the 2023–2024 Omicron XBB.1.5-containing mRNA COVID-19 vaccines [10–14]; however, data on the real-world effectiveness of the mRNA-1273.815 (mRNA-1273 XBB.1.5 containing vaccine) vaccine specifically are limited [15]. This study aimed to evaluate the VE of the mRNA-1273.815 vaccine against COVID-19–related hospitalizations and any medically attended COVID-19 in adults aged 18 years or older.

## METHODS

### Data Sources

This study leveraged electronic health record (EHR) data from the Veradigm Network EHR linked to healthcare claims sourced from Komodo Health spanning 1 March 2020 through 31 December 2023. The EHR data are sourced from ambulatory/outpatient primary care and specialty settings, and the claims data include inpatient, outpatient, and pharmacy sources. This integrated dataset has been previously characterized and used previously in COVID-19 epidemiology and VE research [2, 16–18]. The dataset used in this study contains only deidentified data as per the deidentification standard defined in Section §164.514(a) of the Health Insurance Portability and Accountability Act of 1996 (HIPAA) Privacy Rule. As a noninterventional, retrospective database study using data from a certified HIPAA-compliant deidentified research database, approval by an institutional review board was not required.

### Study Design Overview

**Figure 1.**
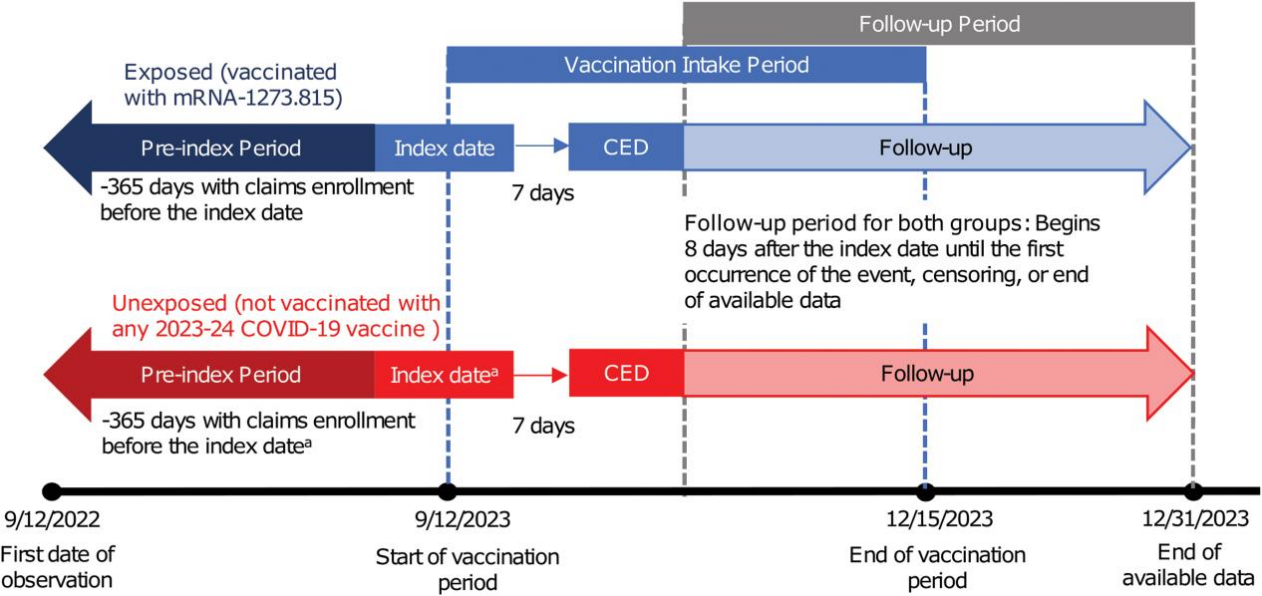
Schematic of the study design with definition of the preindex period, index date, cohort entry date (CED), and follow-up period. ^a^The index date for unexposed individuals was assigned based on their 1:1 match in the exposed cohort.

We conducted a retrospective, noninterventional cohort study of mRNA-1273.815 VE in adults who were aged ≥18 years and residing in the United States. Adults vaccinated with mRNA-1273.815 during the vaccine intake period were identified and matched in a 1:1 ratio to adults with no evidence of receiving a 2023–2024 Omicron XBB.1.5-containing COVID-19 vaccine. Baseline patient characteristics (covariates) were measured, and inverse probability of treatment weighting (IPTW) was used to further reduce potential confounding by creating a weighted study sample based on the predefined covariates (see subsection Covariates). Individuals in the vaccinated (exposed) and unvaccinated (unexposed) cohorts were followed for the 2 key outcomes, COVID-19–related hospitalizations and any medically attended COVID-19, during the follow-up period. Cox regression models were used to calculate hazard ratios (HR) and 95% confidence intervals (95% CI), which were converted to VE estimates. The study design is shown in Figure 1.

### Study Population

Individuals with a record of vaccination with mRNA-1273.815 (50 mcg) during the vaccine intake period (12 September 2023–15 December 2023) were eligible for inclusion in the exposed cohort. Individuals without a record of vaccination with any 2023–2024 updated COVID-19 vaccine during the vaccine intake period were eligible for inclusion in the unexposed cohort.

The index date of the individuals in the exposed cohort was the date of their first mRNA-1273.815 vaccination. Individuals in the unexposed cohort were assigned multiple potential index dates based on the index dates of all possible direct matches in the exposed cohort using the following matching criteria: age on 12 September 2023, sex, race, ethnicity, region, week of last claims or EHR activity, and receipt of a bivalent BA.4/BA.5 COVID-19 vaccine between 1 September 2022 and 11 September 2023.

We required that individuals have continuous enrollment in medical and pharmacy claims from 12 September 2022, through 7 days after the index date (cohort entry date [CED]). Individuals with any of the following were excluded: (1) missing sex or region data, (2) evidence of COVID-19 diagnosis or treatment between 90 days before and 7 days after the index date, or (3) evidence of vaccination with any COVID-19 vaccine in the 60 days before the index day through the CED (excluding the index vaccination in the exposed group). Age was a required field in the underlying data file. Individuals were required to have ≥1 day of follow-up after the CED. Follow-up began the day after the CED and continued until the earliest of the following: outcome of interest, the end of continuous enrollment, receipt of a subsequent COVID-19 vaccine, or end of the follow-up period (31 December 2023).

After these selection criteria were applied, the final matches were created as follows. First, an individual in the exposed cohort was randomly selected. Then, if they had exactly 1 match in the unexposed cohort who met all the selection criteria, they were assigned that individual as a match, and both were removed from further matching. If they had multiple potential matches who met all the selection criteria, they were randomly assigned 1 match, and both were removed from further matching. If the exposed individual had zero remaining matches who met all the selection criteria, they were excluded from the study. This process was repeated until all individuals in the exposed cohort were either matched to someone in the unexposed cohort or excluded from the study. Individuals in the unexposed cohort who were not matched to an individual in the exposed cohort were excluded. The final index date for individuals in the unexposed cohort was the same as their matched mRNA-1273.815 counterpart. By matching on the week of latest activity and then assigning an index date based on their final match, we ensured that follow-up time was similar between the 2 cohorts.

The following subsets of the overall study population were defined for exploratory analyses: adults aged ≥50 years, adults aged ≥65 years, and adults aged ≥18 years with at least 1 underlying medical condition that increased the risk of severe outcomes from COVID-19 as defined by the CDC [17]. The codes used to identify 2023–2024 Omicron XBB.1.5-containing COVID-19 vaccines, any COVID-19 vaccination, and COVID-19 outcomes are reported in Supplementary Tables 1–3.

### Outcome Measures

The primary outcome of interest was COVID-19–related hospitalization, and the secondary outcome of interest was any medically attended COVID-19. Each outcome was evaluated independently so that an individual could be counted as having both a COVID-19–related hospitalization and medically attended COVID-19.

COVID-19–related hospitalizations were identified from hospitalization claims with documentation of COVID-19 diagnosis in any position. Any medically attended COVID-19 included various clinical settings such as inpatient admissions, outpatient visits (emergency department visits, urgent care visits, office visits, and telemedicine visits), and laboratory results. These were identified from an EHR or medical claim with either a COVID-19 diagnosis in any setting or a positive laboratory test result in the ambulatory care setting. This may include positive test results for asymptomatic infections. The codes used to identify COVID-19 diagnosis are reported in Supplementary Table 3.

### Covariates

The study used covariate information including demographics (age on 12 September 2023, sex [female, male], race [Black, White, other, and unknown], ethnicity [Hispanic, non-Hispanic, and unknown]), insurance type (commercial, Medicaid, Medicare Advantage, Medicare Fee-For-Service, other, and unknown), region (Midwest, Northeast, South, West, and unknown), and month of index. Time since last COVID-19 vaccination and time since last COVID-19 infection were captured using a look-back period that started on 1 March 2020. The number of outpatient visits and the number of hospitalizations were captured using a 12-month preperiod.

The presence of underlying medical conditions associated with an increased risk of severe COVID-19 outcomes, as defined by the US CDC, was captured [19]. These included asthma, cancer, cerebrovascular disease, chronic kidney disease, chronic liver disease, chronic lung disease, cystic fibrosis, dementia, diabetes mellitus, disability, heart conditions, HIV, mental health conditions, obesity (body mass index > 30), physical inactivity, pregnancy, primary immunodeficiencies, respiratory tuberculosis, smoking, and solid organ or stem cell transplant, and use of select immunosuppressive medications. Pregnancy was captured in the 301 days preceding the index date. Other underlying medical conditions were captured in the 12-month preindex period. Code sets and medication lists used to identify underlying medical conditions were previously reported in Kopel et al [2]. Patients with at least 1 of these medical conditions were considered “high risk” for severe COVID-19 outcomes.

### Data Analysis

Propensity scores predicting receipt of mRNA-1273.815 were calculated for each subject using a multivariable logistic model adjusted for all covariates outlined above. Stabilized and truncated weights were used to reweight the study sample using the IPTW methodology. Sample balance before and after IPTW was assessed by calculation of the standardized mean differences (SMD). SMDs with absolute values >0.1 indicated covariate imbalance. Covariates were reported descriptively before and after weighting with means and standard deviations for continuous variables and N and percent for categorical variables.

Incidence rates, Kaplan-Meier curves, and unadjusted HRs were reported for the unweighted sample, and adjusted HRs were reported for the weighted sample. Unadjusted HRs were calculated using a Cox regression model with mRNA-1273.815 vaccination as the only predictor. Adjusted HRs were calculated for the weighted sample using a weighted Cox regression model that included mRNA-1273.815 vaccination status and any covariates with an SMD greater than 0.1 after IPTW. Subsequently, the unadjusted and adjusted VE for each outcome were calculated as 100 × (1 – HR) and reported with 95% CIs.

This analysis was conducted using SAS V9.4.

## RESULTS

**Figure 2.**
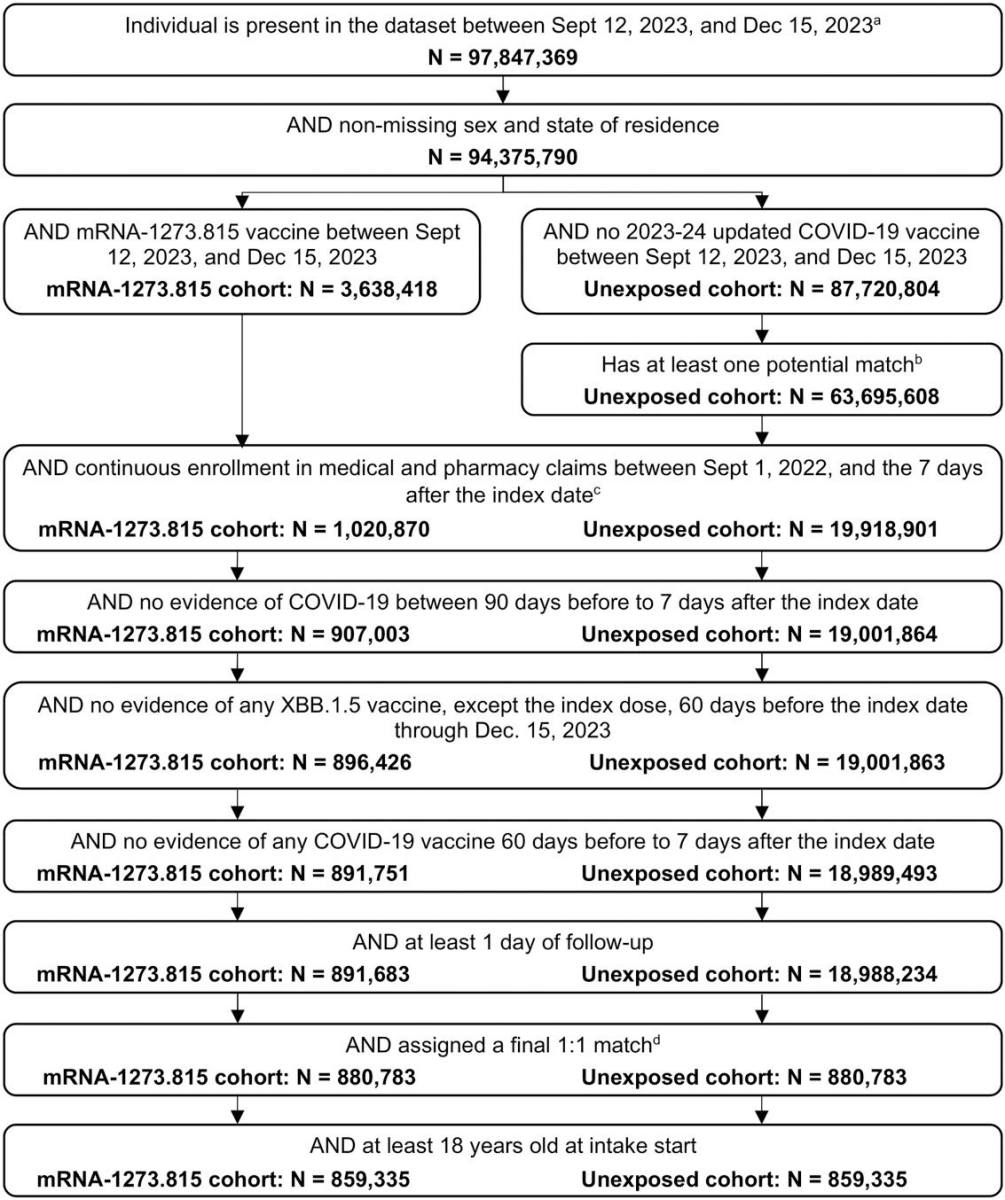
Selection criteria for identification of individuals vaccinated with mRNA-1273.815 and direct matched unexposed individuals. EHR, electronic health record. ^a^Includes individuals with continuous enrollment for any portion of this period along with those who have at least 1 record in claims or EHR during this period. ^b^Matched on age on 12 September 2023, sex, race, ethnicity, region, latest activity week, and receipt of a bivalent COVID-19 vaccine between 1 September 2022 and 11 September 2023. ^c^For the mRNA-1273.815 cohort, the index date is the date of first evidence of mRNA-1273.815 vaccine. For the unexposed cohort, each patient was assigned potential index dates for evaluation based on their potential matches in the mRNA-1273.815 vaccine cohort in line 4. Their final index date was assigned based on their match in line 10. ^d^Among all potential unexposed cohort matches who made it through the waterfall, each individual in the mRNA-1273.815 cohort was randomly assigned exactly 1 final match. Individuals in the mRNA-1273.815 cohort who no longer had any potential matches in the unexposed cohort were excluded. Unmatched unexposed individuals were excluded.

This study included 1 718 670 individuals; 859 335 adults who received the mRNA-1273.815 vaccine between 12 September 2023 and 15 December 2023 (exposed cohort), met all the study inclusion criteria, and were directly matched 1:1 to an unexposed adult (Figure 2). Of these, most had documentation of previous vaccination and some had documentation of previous infection; notably, approximately 70% (N = 605 816) of each cohort had previously received the COVID-19 bivalent vaccine and >85% had a record of previous COVID-19 vaccination.

The exposed and unexposed cohorts were well balanced after weighting, with no SMDs greater than 0.1. After weighting, the mean (standard deviation) age in both cohorts was 63 (16) years; 57% were female, 60% were White, 6% were Black, and more than 80% received their most recent COVID-19 vaccine more than 180 days before the index date (Table 1). Approximately 80% (N = 686 135) of the study population was at least 50 years old and 54% (N = 465 061) were at least 65 years old (Supplementary Table 4).

**Table 1.** Baseline Characteristics of Individuals Included as Covariates in the Main Analysis of the Primary and Secondary Objectives—Data Are Presented as n (%) Unless Otherwise Stated

		Preweighting			Postweighting		
		mRNA-1273.815	Unexposed	SMD	mRNA-1273.815	Unexposed	SMD
Number of patients		859 335	859 335		855 567	858 518	
Age at intake, mean (SD)		63 (16.3)	63 (16.3)	0	63 (16.2)	63 (16.3)	0.003
Sex, N (%)	Female	490 534 (57.1%)	490 534 (57.1%)	0	487 795 (57.0%)	489 828 (57.1%)	<0.001
	Male	368 801 (42.9%)	368 801 (42.9%)		367 772 (43.0%)	368 690 (42.9%)	
Race, N (%)	Black	50 444 (5.9%)	50 444 (5.9%)	0	50 431 (5.9%)	50 585 (5.9%)	0.002
	Other	129 943 (15.1%)	129 943 (15.1%)		128 936 (15.1%)	129 625 (15.1%)	
	White	519 496 (60.5%)	519 496 (60.5%)		518 019 (60.5%)	519 255 (60.5%)	
	Unknown	159 452 (18.6%)	159 452 (18.6%)		158 181 (18.5%)	159 054 (18.5%)	
Ethnicity, N (%)	Hispanic	19 228 (2.2%)	19 228 (2.2%)	0	19 161 (2.2%)	19 230 (2.2%)	<0.001
	Non-Hispanic	506 284 (58.9%)	506 284 (58.9%)		504 390 (59.0%)	506 042 (58.9%)	
	Unknown	333 823 (38.8%)	333 823 (38.8%)		332 016 (38.8%)	333 247 (38.8%)	
Insurance type^a^, N (%)	Commercial	359 650 (41.9%)	331 467 (38.6%)	0.252	345 687 (40.4%)	345 777 (40.3%)	0.014
	Medicaid	45 032 (5.2%)	103 319 (12.0%)		70 771 (8.3%)	74 236 (8.6%)	
	Medicare Advantage	346 959 (40.4%)	316 761 (36.9%)		331 133 (38.7%)	330 519 (38.5%)	
	Medicare FFS	7486 (0.9%)	12 455 (1.4%)		9825 (1.1%)	9960 (1.2%)	
	Other	96 309 (11.2%)	90 716 (10.6%)		93 889 (11.0%)	93 764 (10.9%)	
	Unknown	3899 (0.5%)	4617 (0.5%)		4262 (0.5%)	4263 (0.5%)	
Region, N (%)	Midwest	201 209 (23.4%)	201 209 (23.4%)	0	200 289 (23.4%)	200 933 (23.4%)	<0.001
	Northeast	212 504 (24.7%)	212 504 (24.7%)		211 548 (24.7%)	212 299 (24.7%)	
	South	261 628 (30.4%)	261 628 (30.4%)		260 827 (30.5%)	261 549 (30.5%)	
	West	183 978 (21.4%)	183 978 (21.4%)		182 887 (21.4%)	183 722 (21.4%)	
	Unknown	16 (0.0%)	16 (0.0%)		16 (0.0%)	16 (0.0%)	
Month of index, N (%)	September 2023	131 504 (15.3%)	131 504 (15.3%)		127 961 (15.0%)	132 666 (15.5%)	
	October 2023	435 505 (50.7%)	435 505 (50.7%)		431 090 (50.4%)	435 894 (50.8%)	
	November 2023	230 482 (26.8%)	230 482 (26.8%)		233 467 (27.3%)	228 676 (26.6%)	
	December 2023	61 844 (7.2%)	61 844 (7.2%)		63 050 (7.4%)	61 283 (7.1%)	
Number of outpatient visits, mean (SD)		2.5 (6.1)	2.8 (6.7)	0.046	2.6 (6.5)	2.6 (6.4)	<0.001
Number of hospitalizations, mean (SD)		0.1 (0.5)	0.2 (0.8)	0.111	0.2 (0.7)	0.2 (0.7)	0.010
Time since last COVID-19 vaccine, N (%)	60- ≤ 90 d	4294 (0.5%)	8279 (1.0%)	0.233	6229 (0.7%)	6309 (0.7%)	0.012
	91–180 d	48 897 (5.7%)	33 964 (4.0%)		41 057 (4.8%)	40 557 (4.7%)	
	>180 d	729 900 (84.9%)	680 131 (79.1%)		705 358 (82.4%)	705 288 (82.2%)	
	Not reported	76 244 (8.9%)	136 961 (15.9%)		102 924 (12.0%)	106 364 (12.4%)	
Time since last COVID-19 infection, N (%)	≤120 d	2773 (0.3%)	2547 (0.3%)	0.092	2650 (0.3%)	2657 (0.3%)	0.004
	121–180 d	4659 (0.5%)	4453 (0.5%)		4529 (0.5%)	4550 (0.5%)	
	>180 d	107 547 (12.5%)	135 113 (15.7%)		119 848 (14.0%)	121 310 (14.1%)	
	Not reported	744 356 (86.6%)	717 222 (83.5%)		728 540 (85.2%)	730 002 (85.0%)	
Patients with underlying medical conditions, N (%)	Asthma	62 889 (7.3%)	65 534 (7.6%)	0.012	64 149 (7.5%)	64 388 (7.5%)	<0.001
	Cancer	72 288 (8.4%)	76 451 (8.9%)	0.017	74 348 (8.7%)	74 496 (8.7%)	<0.001
	Cerebrovascular disease	47 659 (5.5%)	61 963 (7.2%)	0.068	54 323 (6.3%)	54 966 (6.4%)	0.002
	Chronic kidney disease	79 032 (9.2%)	96 586 (11.2%)	0.068	87 441 (10.2%)	88 088 (10.3%)	0.001
	Chronic lung disease	65 155 (7.6%)	85 061 (9.9%)	0.082	74 755 (8.7%)	75 485 (8.8%)	0.002
	Chronic liver disease	8636 (1.0%)	11 492 (1.3%)	0.031	10 003 (1.2%)	10 135 (1.2%)	0.001
	Cystic fibrosis	134 (0.0%)	128 (0.0%)	<0.001	128 (0.0%)	129 (0.0%)	<0.001
	Diabetes type 1 or 2	173 719 (20.2%)	204 775 (23.8%)	0.087	188 907 (22.1%)	189 939 (22.1%)	0.001
	Disability	49 050 (5.7%)	50 148 (5.8%)	0.006	49 885 (5.8%)	49 966 (5.8%)	<0.001
	Heart conditions	124 732 (14.5%)	151 068 (17.6%)	0.084	137 084 (16.0%)	138 141 (16.1%)	0.002
	HIV	3665 (0.4%)	4001 (0.5%)	0.006	3852 (0.5%)	3863 (0.4%)	<0.001
	Mental health disorders	123 072 (14.3%)	142 194 (16.5%)	0.062	132 719 (15.5%)	133 482 (15.5%)	0.001
	Neurological conditions	20 390 (2.4%)	33 239 (3.9%)	0.086	26 394 (3.1%)	26 993 (3.1%)	0.003
	Obesity	193 592 (22.5%)	215 532 (25.1%)	0.060	204 329 (23.9%)	205 193 (23.9%)	<0.001
	Primary immunodeficiencies	10 061 (1.2%)	11 991 (1.4%)	0.020	11 051 (1.3%)	11 077 (1.3%)	<0.001
	Pregnancy	1189 (0.1%)	1316 (0.2%)	0.004	1203 (0.1%)	1239 (0.1%)	0.001
	Physical inactivity	616 (0.1%)	856 (0.1%)	0.010	734 (0.1%)	743 (0.1%)	<0.001
	Smoking	96 971 (11.3%)	122 476 (14.3%)	0.089	109 043 (12.7%)	110 116 (12.8%)	0.002
	Solid organ or hematopoietic stem cell transplant	2583 (0.3%)	2934 (0.3%)	0.007	2802 (0.3%)	2796 (0.3%)	<0.001
	Tuberculosis	197 (0.0%)	301 (0.0%)	0.007	247 (0.0%)	252 (0.0%)	<0.001
	Use of immunosuppressants	42 261 (4.9%)	39 729 (4.6%)	0.014	41 128 (4.8%)	41 049 (4.8%)	0.001

Abbreviations: FFS, fee for service; SD, standard deviation; SMD, standardized mean difference.

^a^Payers were evaluated in this order: Medicare FFS -> Medicare Advantage -> Medicaid -> Commercial -> Other -> Unknown. Individuals who were dual eligible for Medicare and Medicaid were counted as Medicare for the purpose of this study.

Preweighting, the majority of individuals, including 61.5% (N = 528 465) of the mRNA-1273.815 cohort and 66.4% (N = 570 783) of the unexposed cohort, had at least 1 underlying medical condition associated with an increased risk of severe COVID-19 outcomes (Supplementary Table 4). The most common underlying medical conditions preweighting were obesity (exposed: 22.5% and unexposed: 25.1%), diabetes mellitus (20.2% and 23.8%, respectively), and heart conditions (14.5% and 17.6%, respectively) (Table 1).

The overall median (interquartile range) follow-up time was 63 (44–78) days. The median follow-up time for the age ≥50 years, age ≥ 65 years, and high-risk cohorts were 64, 65, and 63 days, respectively (Supplementary Table 4). For the overall population, 794 COVID-19–related hospitalizations and 8710 COVID-19–related medical encounters were identified preweighing. Of these, 88.2% of hospitalizations and 67.4% of COVID-19–related medical encounters occurred among adults aged at least 65 years. For reference, counts of COVID-19–related outpatient encounters and positive laboratory tests for COVID-19 are reported in Supplementary Table 4.

**Figure 3.**
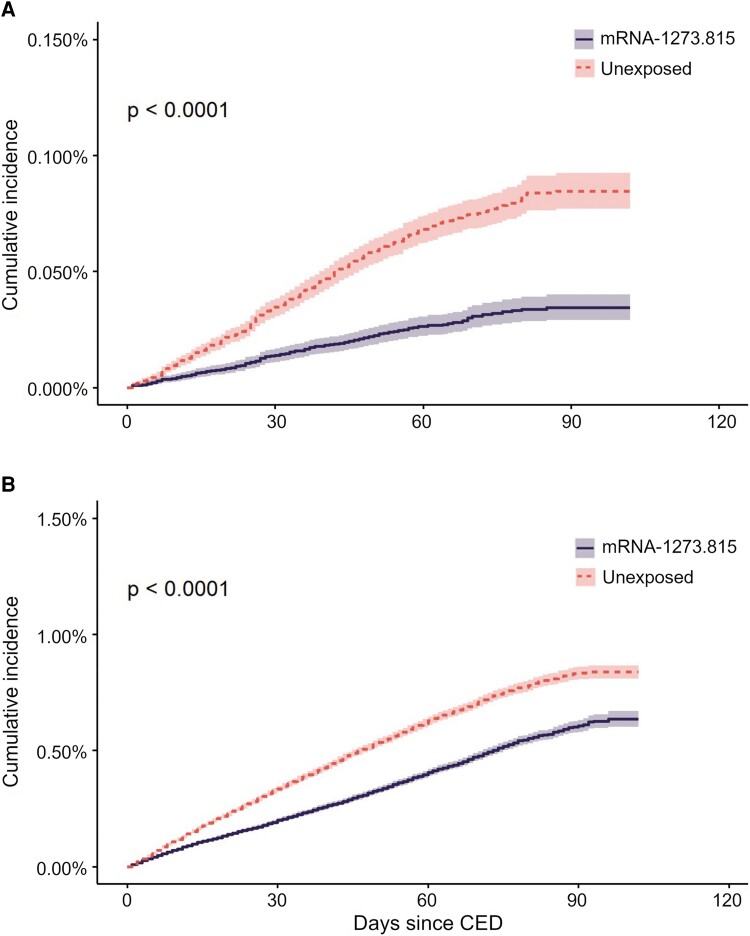
Cumulative incidence of (*A*) COVID-19–related hospitalizations and (*B*) any medically attended COVID-19 between the cohort entry date (CED) and 31 December 2023, among individuals vaccinated with mRNA-1273.815 and direct matched unexposed individuals.

The incidence rate of COVID-19 hospitalizations per 100 000 per week was 7.38 (95% CI, 6.80–7.99) in the unexposed cohort and 2.32 (95% CI, 2.01–2.68) in the exposed cohort throughout the entire follow-up period. The incidence rate of any medically attended COVID-19 per 100 000 per week was 65.18 (95% CI, 63.44–66.95) in the unexposed cohort and 41.44 (95% CI, 40.05–42.85) in the exposed cohort. Kaplan-Meier curves of outcomes show that the incidence rate was fairly steady through the follow-up period (Figure 3).

**Figure 4.**
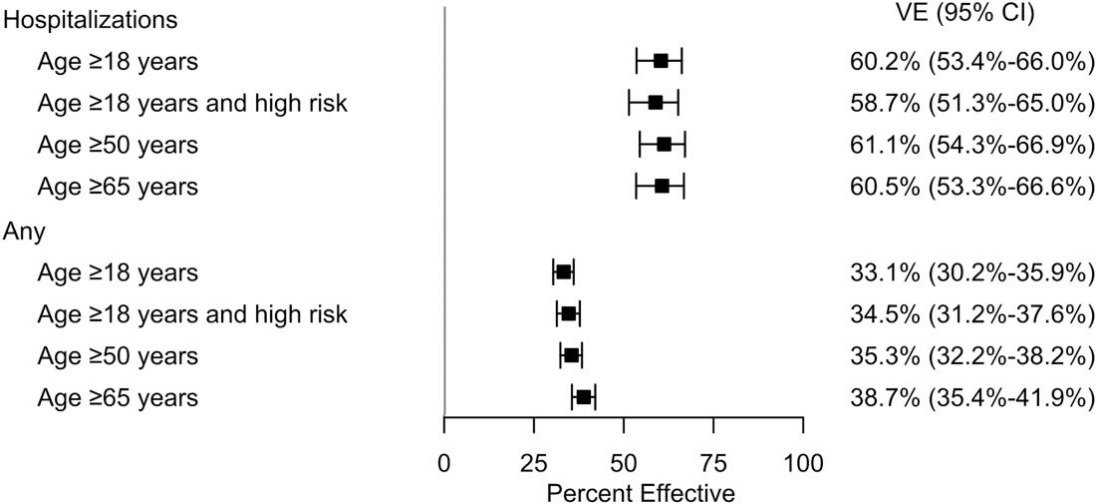
Adjusted vaccine effectiveness (VE) estimates with 95% confidence intervals (CI) of the mRNA-1273.815 COVID-19 vaccine (reference: unexposed cohort), September 2023–December 2023. *Defined by Centers for Disease Control and Prevention: https://www.cdc.gov/covid/hcp/clinical-care/underlying-conditions.html.

The adjusted VE (95% CI) against COVID-19–related hospitalizations was 60.2% (53.4–66.0) among adults at least 18 years old (Figure 4). Similarly, the VE against COVID-19–related hospitalizations was 61.1% (54.3–66.9) among adults ≥50 years of age, 60.5% (53.3–66.6) among adults ≥65 years of age, and 58.7% (51.3–65.0) among adults with underlying medical conditions.

The adjusted VE against any medically attended COVID-19 was 33.1% (30.2–35.9) among adults at least 18 years old. VE against medically attended COVID-19 was 35.3% (32.2–38.2) for adults ≥50 years old, 38.7% (35.4–41.9) among adults ≥65 years old, and 34.5% (31.2–37.6) among adults with at least 1 underlying medical condition. Unweighted, unadjusted VE estimates are presented in Supplementary Table 5.

## DISCUSSION

In this analysis of 1 718 670 US adults aged ≥18 years, vaccination with mRNA-1273.815, an Omicron XBB.1.5-containing COVID-19 vaccine, was associated with significant protection against COVID-19–related hospitalizations and medically attended COVID-19. Notably, the significant effectiveness of mRNA-1273.815 was observed in all subgroups, including older adults and those with specific underlying medical conditions that may increase their risk of severe COVID-19. These findings support CDC recommendations to remain up to date with COVID-19 vaccination and underscore the benefit of receiving mRNA1273.815 for the general population and those at higher risk for COVID-19–related morbidity and mortality.

These findings are consistent with other real-world effectiveness studies of 2023–2024 Omicron XBB.1.5-containing COVID-19 vaccines. Initial effectiveness studies conducted in Denmark and the Netherlands, primarily with the BNT162b2 XBB.1.5-adapted vaccine, reported effectiveness estimates of greater than 70% against COVID-19–related hospitalizations over a short follow-up period [11, 12]. Real-world studies conducted in the United States estimated the effectiveness of the 2023–2024 COVID-19 vaccines to be between ∼40% and 60% for COVID-19 outcomes, including symptomatic infection, illness resulting in an emergency department/urgent care visit, or hospitalization with a median follow-up period of 30–50 days [10, 13, 14, 20]. Although these studies differ in methods, population, outcomes, and duration, they are consistent with this study's findings and demonstrate the significant benefit of vaccination with the updated 2023–2024 COVID-19 vaccines. Furthermore, the follow-up postvaccination in this current study is one of the longest, exceeding 60 days. As the circulating variants continue to evolve, additional analyses are needed to assess the duration of the effectiveness over time.

The results of this study are also consistent with neutralizing antibody data generated among individuals who received mRNA-1273.815. A study in which 50 individuals received mRNA-1273.815 demonstrated a 17.5-fold increase in neutralizing antibody levels against XBB.1.5 at day 29 postvaccination relative to prevaccination antibody titers [9]. A similar increase was observed against other SARS-CoV-2 variants, including EG.5.1, XBB.1.16, BA.4/BA.5, EG.5.1, BA.2.86, and JN.1, supporting significant cross-reactive immune responses from 2023–2024 Omicron XBB.1.5-containing vaccination across related SARS-CoV-2 variants. Data from a US CDC IVY study assessing the effectiveness of the updated 2023–2024 COVID-19 vaccines reported that, among the >900 samples that were sequenced from SARS-CoV-2–positive specimens in the study, only 16% had an XBB.1.5-like spike protein, whereas almost 80% had spike proteins of other XBB lineage variants and ∼5% had JN.1-like spike proteins [13]. These data suggest that the Omicron XBB.1.5-containing vaccine formulation offers protection from a range of XBB variants, including newer variants in the XBB lineage, like EG.5 and HK.3.

Four years since the start of the COVID-19 pandemic, a significant portion of the adult population in the United States has developed immune responses against the SARS-CoV-2 virus, whether through previous infections, vaccinations, or a combination of both [21]. This widespread seropositivity offers a degree of immunity within the community, which tends to diminish in the face of new, evolving virus variants. Although the current study included individuals regardless of their vaccination and infection history, the majority of the population in both cohorts received the bivalent vaccine and cohort adjustment included vaccination and infection history. Thus, the results of this study highlight the incremental protection provided by the additional dose targeting the circulating variants in a real-world setting regardless of exposure and/or vaccination history.

The majority of the US adult population has completed their primary series vaccination. However, as of May 2024, fewer than 1 in 4 adults aged ≥18 years and approximately 40% of adults aged ≥65 years had received a 2023–2024 updated COVID-19 vaccine [22]. Studies that include individuals who were previously vaccinated or infected and show the incremental protection provided by updated vaccines, especially against severe disease, are essential and should be routinely communicated to increase vaccine confidence among healthcare providers and the general population.

### Limitations

Study limitations include a lack of generalizability to uninsured individuals or those with less stable insurance, as individuals were required to have continuous enrollment in claims. In addition, inherent to most noninterventional studies using real-world data, selection bias may be present due to the nonrandomized nature of data collection. To mitigate this, direct 1:1 matching and propensity score weighting were conducted using a broad range of covariates. After IPTW, balance was achieved on all measured characteristics. However, the possibility of residual unmeasured confounding persists because there may be characteristics not accounted for with the chosen study covariates, and some included covariates, such as time since last COVID-19 infection, might not be well captured in the data source if patients used an at-home test and never sought medical attention for their infection. Additionally, IPTW cannot account for differences in risk-taking behavior between the 2 cohorts. Uptake of the XBB.1.5-containing vaccines was lower than uptake of prior COVID-19 vaccines, and individuals who received an XBB.1.5-containing vaccine may be more aware of the risks of COVID-19 and more likely to take other steps to mitigate the risk of COVID-19 infection.

Furthermore, like all retrospective database studies, this study is subject to limitations because of data completeness. For example, the absence of a vaccination record does not guarantee that a patient did not receive a vaccine. Although we cannot determine the degree of missingness, this study leveraged a linked database enriched with open claims sources, closed claims sources, and EHR sources for the identification of exposures, covariates, and outcomes during a period of continuous claims enrollment, which reduces but does not eliminate the risk of missingness. Since the COVID-19 vaccine was commercialized during the 2023–2024 respiratory season and not distributed under an emergency use authorization, it was more likely that documentation of payment would be included in the claims dataset. Furthermore, by requiring documentation of age, sex, and state of residence and matching on criteria like prior COVID-19 vaccination, we reduced the risk of bias in missingness between the 2 cohorts. In addition, outcomes identification in the dataset may be limited in terms of specificity and sensitivity. For example, any medically attended COVID-19 could potentially include asymptomatic infections, which resulted in a positive COVID-19 test, but would exclude infections that resulted in a positive at-home COVID-19 infection for which the individual never sought medical care.

In this analysis, we were not able to differentiate between hospitalization “due to” versus hospitalization “with” COVID-19. The selected hospitalization outcome was based on a validation study of several US claims data sources in 2020, estimating the positive predictive value of the U07.1 diagnostic code alone and in any position to range from 94.1% to 81.2% for identifying COVID-19 hospitalizations [23]. This approach is consistent with prior publications evaluating the effectiveness of COVID-19 vaccines [18, 24]. Notably, the robustness of the dataset and completeness of results are further supported by the hospitalization incidence rates in this study (unexposed group), which were consistent with those reported by the CDC [3].

Last, several factors may contribute to an over- or underestimate of VE. For example, we used a 7-day window between vaccination and the cohort entry data, which may result in capturing some infections among vaccinated individuals before the development of a full immune response. This approach is consistent with other studies of the XBB.1.5 updated COVID-19 vaccines but may result in an underestimation of VE [11, 12]. On the other hand, although SARS-CoV-2 still does not have a clear seasonality, this analysis included only part of the winter season, which is usually characterized by an increase in incidence rates [25]. Partial season analyses, similar to the study presented here, aim to inform vaccine recommendations in a timely manner but are limited in scope to the time period when VE is likely highest. Vaccine effectiveness is anticipated to wane with increasing time between vaccination and virus exposure and with the evolution of the circulation of new variants. Thus, although this study provides a follow-up duration of more than 60 days, as SARS-CoV-2 continues to evolve, further analyses are needed to determine the durability of protection.

## CONCLUSION

In this real-world effectiveness assessment, the mRNA-1273.815 vaccine (2023–2024 Omicron XBB.1.5-containing mRNA COVID-19 vaccine) provided significant protection against COVID-19-related hospitalizations and medically attended COVID-19. These findings are consistent with effectiveness estimates of 2023–2024 updated COVID-19 vaccines overall, demonstrating the incremental value over earlier vaccinations and the continued role of updated COVID-19 vaccinations in protection against COVID-19–related outcomes.

## Supplementary Material

ofae695_Supplementary_Data

## Supplementary Data

Supplementary materials are available at *Open Forum Infectious Diseases* online. Consisting of data provided by the authors to benefit the reader, the posted materials are not copyedited and are the sole responsibility of the authors, so questions or comments should be addressed to the corresponding author.

## Notes

***Acknowledgments***. Christopher Adams, an employee of Veradigm, provided programming support and quality assurance for this analysis. This support was funded via a contract with Moderna.

***Author contributions***. Conceptualization: J.A.M., H.K., A.B.A., D.M., M.A.M., T.L., and V.H.N. Methodology: all authors. Software: N.Z. Validation: A.B., J.P.W.-J., N.Z., and I.H.W. Formal analysis: A.B., J.P.W.-J., N.Z., and I.H.W. Investigation: A.B., J.P.W.-J., N.Z., and I.H.W. Resources: M.B. Data curation: N.Z. Writing—original draft preparation: J.P.W.-J., J.A.M., and H.K. Writing—review and editing: all authors. Visualization: J.P.W.-J., J.A.M., and H.K. Supervision: A.B., J.A.M., D.M., H.K., and M.B. Project administration: A.B. and M.B. Funding acquisition: J.A.M., D.M., H.K., and M.B. All authors have read and agreed to the published version of the manuscript.

***Financial support***. This work was supported by Moderna, Inc. via a contract with Veradigm.

***Data availability statement***. The data that support the findings of this study were used under license from Veradigm and Komodo Health. Because of data use agreements and their proprietary nature, restrictions apply regarding the availability of the data. Further information is available from the corresponding author.

***Ethics approval and consent to participate***. The linked dataset only contains deidentified data as per the deidentification standard defined in Section §164.514(a) of the Health Insurance Portability and Accountability Act of 1996 (HIPAA) Privacy Rule. The process by which the data are deidentified is attested to through a formal determination by a qualified expert as defined in Section §164.514(b)(1) of the HIPAA Privacy Rule. Because this study used only deidentified patient records, it is therefore no longer subject to the HIPAA Privacy Rule and is therefore exempt from institutional review board approval and for obtaining informed consent according to US law. This study was conducted in compliance with the Declaration of Helsinki and used only deidentified data.
