# Supplementary material for: Effectiveness of the 2023–2024 Omicron XBB.1.5-containing mRNA COVID-19 Vaccine (mRNA-1273.815) in Preventing COVID-19–related Hospitalizations and Medical Encounters Among Adults in the United States
Source: Open Forum Infect Dis. 2024 Nov 26;11(12):ofae695. doi: 10.1093/ofid/ofae695 (PMC11651145; doi:10.1093/ofid/ofae695)
Supplement: ofae695_Supplementary_Data [file ofae695_supplementary_data.docx]

**Supplementary Tables for Effectiveness of the 2023-2024 Omicron XBB.1.5-containing mRNA COVID-19 vaccine (mRNA-1273.815) in preventing COVID-19-related hospitalizations and medical encounters among adults in the United States**

Supplementary Table 1. List of codes used to identify 2023-2024 Omicron XBB.1.5-containing COVID-19 vaccines from the Veradigm EHR and linked claims datasets.

| **COVID-19 vaccine type** | **CPT** | **CVX** | **NDC** |
| --- | --- | --- | --- |
| mRNA-1273.815 | 91321, 91322 | 311, 312 | 80777-0102-01, 80777-0102-04, 80777-0102-93, 80777-0102-95, 80777-0102-96, 80777-0287-07, 80777-0287-92 |
| BNT162b2 XBB.1.5-adapted vaccine | 91318, 91319, 91320 | 308, 309, 310 | 00069-2362-01, 00069-2362-10, 00069-2392-01, 00069-2392-10, 59267-4315-01, 59267-4315-02, 59267-4331-01, 59267-4331-02 |
| 2023-2024 Novavax updated vaccine |  |  | 80631-0105-01, 80631-0105-02 |

CPT, Current Procedural Terminology; CVX, vaccine administered codes; NDC, National Drug Codes

Supplementary Table 2. List of CVX, CPT, and NDC codes used to identify COVID-19 vaccines from the Veradigm EHR and linked claims datasets.

| **Manufacturer** | **CPT** | **CVX** | **NDC** | **ICD-10-PCS** |
| --- | --- | --- | --- | --- |
| AstraZeneca | 91302, 0021A, 0022A | 210 | 00310-1222-10, 00310-1222-15 |  |
| Janssen | 91303, 0031A, 0034A | 212 | 59676-0580-05, 59676-0580-15 |  |
| Moderna | 91301, 91306, 91309, 91311, 91313, 91314, 91316, 91321, 91322, 0011A, 0012A, 0013A, 0064A, 0091A, 0092A, 0093A, 0094A, 0111A, 0112A, 0113A, 0134A, 0141A, 0142A, 0144A, 0164A | 207, 221, 227, 228, 229, 230, 311, 312, 519 | 61434-0043-02, 80777-0100-11, 80777-0100-15, 80777-0100-98, 80777-0100-99, 80777-0102-01, 80777-0102-04, 80777-0102-93, 80777-0102-95, 80777-0102-96, 80777-0273-10, 80777-0273-15, 80777-0273-98, 80777-0273-99, 80777-0275-05, 80777-0275-99, 80777-0277-05, 80777-0277-99, 80777-0279-05, 80777-0279-99, 80777-0280-05, 80777-0280-99, 80777-0282-05, 80777-0282-99, 80777-0283-02, 80777-0283-99, 80777-0287-07, 80777-0287-92 |  |
| Novavax | 91304, 0041A, 0042A, 0044A | 211, 313 | 80631-0100-01, 80631-0100-10, 80631-0102-01, 80631-0105-01, 80631-0105-02, 80631-1000-01 |  |
| Pfizer | 91317, 91318, 91319, 91320, 0121A, 0151A, 0171A, 0172A, 0173A , 91300, 91305, 91307, 91308, 91312, 91315, 0001A, 0002A, 0003A, 0004A, 0051A, 0052A, 0053A, 0054A, 0071A, 0072A, 0073A, 0074A, 0081A, 0082A, 0083A, 0124A, 0154A | 219, 300, 301, 302, 308, 309, 310, 520, 208, 217, 218 | 00069-1000-01, 00069-1000-02, 00069-1000-03, 00069-2025-01, 00069-2025-10, 00069-2025-25, 00069-2362-01, 00069-2362-10, 00069-2392-01, 00069-2392-10, 59267-0078-01, 59267-0078-02, 59267-0078-04, 59267-0304-01, 59267-0304-02, 59267-0565-01, 59267-0565-02, 59267-0609-01, 59267-0609-02, 59267-1000-01, 59267-1000-02, 59267-1000-03, 59267-1025-01, 59267-1025-02, 59267-1025-03, 59267-1025-04, 59267-1055-01, 59267-1055-02, 59267-1055-04, 59267-1404-01, 59267-1404-02, 59267-4315-01, 59267-4315-02, 59267-4331-01, 59267-4331-02 |  |
| Sanofi | 91310, 0104A | 225, 226 | 49281-0618-20, 49281-0618-78 |  |
| Not Specified | M0201 | 213 |  | XW013S6, XW013T6, XW013U6, XW023S6, XW023T6, XW023U6 |

CPT, Current Procedural Terminology; CVX, vaccine administered codes; ICD-10-PCS, International Classification of Disease, 10^th^ edition, Procedure Coding System; NDC, National Drug Codes

Supplementary Table 3. Codes used to identify COVID-19 diagnosis or treatment

| **Category** | **Code Type** | **Codes for COVID-related medical encounters** |
| --- | --- | --- |
| COVID-19 treatment | Generic drug name | abatacept, anakinra, bamlanivimab, baricitinib, bebtelovimab, betamethasone, budesonide, bupivacaine/dexamethasone, casirivimab/imdevimab, cortisone acetate, deflazacort, dexamethasone, hydrocortisone, infliximab, methylprednisolone, molnupiravir, nirmatrelvir/ritonavir, prednisolone, prednisone, remdesivir, sotrovimab, tocilizumab, triamcinolone, vilobelimab |
| COVID-19 treatment | HCPCS | M0245, M0246, Q0245, M0222, M0223, Q0222, M0240, M0241, M0243, M0244, Q0240, Q0243, Q0244, J0248, M0247, M0248, Q0247, J3262, M0249, M0250, Q0249, J0129, J1745, Q5102, Q5103, Q5104, Q5109, Q5121, C9469, J0702, J1020, J1030, J1040, J1094, J1100, J1700, J1710, J1720, J2650, J2920, J2930, J3300, J3301, J3302, J3303, J3304, J7509, J7510, J8540, Q9993 |
| COVID-19 treatment | ICD-10-PCS | XW033F6, XW043F6, XW033E5, XW043E5, XW033H5, XW043H5 |
| COVID-19 diagnosis | ICD-10-CM | J1282, U071, U072, B34.2 |
| COVID-19 diagnosis | SNOMED | 1119302008, 119731000146105, 119741000146102, 119751000146104, 119981000146107, 1240411000000107, 1240521000000100, 1240531000000103, 1240541000000107, 1240561000000108, 1240581000000104, 674814021000119106, 840533007, 840534001, 840536004, 840539006, 866151004, 866152006, 870577009, 870588003, 870589006, 870590002, 870591003, 871562009 |

CPT, Current Procedural Terminology; HCPCS, Healthcare Common Procedure Coding System; ICD-10-PCS, International Classification of Disease, 10^th^ edition, Procedure Coding System; ICD-10-CM, International Classification of Disease, 10^th^ edition, Clinical Modification, SNOMED, Systemized Nomenclature of Medicine

**Supplementary Table 4.**  **Population and Event Counts During the Variable Follow-up Period**

|  | All adults (≥18 years) | | ≥18 years and high risk^a^ | | ≥50 years | | ≥65 years | |
| --- | --- | --- | --- | --- | --- | --- | --- | --- |
|  | mRNA-1273.815 | unexposed | mRNA-1273.815 | unexposed | mRNA-1273.815 | unexposed | mRNA-1273.815 | unexposed |
| Population counts, pre-IPTW | 859,335 | 859,335 | 528,465 | 570,783 | 686,135 | 686,135 | 465,061 | 465,061 |
| Population counts, post-IPTW | 855,567 | 858,518 | 526,294 | 570,226 | 683,370 | 685,233 | 463,172 | 464,267 |
| Follow-up time in days, median (IQR) | 63 (44–78) | 63 (44–78) | 64 (45–78) | 63 (44–77) | 64 (45–78) | 64 (45–78) | 65 (46–79) | 65 (46–79 |
| Any medically-attended COVID-19, counts (%), pre-IPTW | 3,399 (0.4%) | 5,311 (0.6%) | 2,624 (0.5%) | 4,429 (0.8%) | 2,946 (0.4%) | 4,748 (0.7%) | 2,149 (0.5%) | 3,718 (0.8%) |
| COVID-19-related hospitalization, counts (%), pre-IPTW | 191 (0.02%) | 603 (0.07%) | 178 (0.03%) | 557 (0.10%) | 182 (0.03%) | 584 (0.09%) | 167 (0.04%) | 533 (0.11%) |
| COVID-19-related outpatient medical encounters, counts (%), pre-IPTW | 3,275 (0.4%) | 4,971 (0.6%) | 2,510 (0.5%) | 4,123 (0.7%) | 2,831 (0.4%) | 4,421 (0.6%) | 2,044 (0.4%) | 3,421 (0.7%) |
| Positive lab test for COVID-19^b^, counts (%), pre-IPTW | 14 (<0.01%) | 24 (<0.01%) | 7  (<0.01%) | 22 (<0.01%) | 11 (<0.01%) | 22 (<0.01%) | 9  (<0.01%) | 16 (<0.01%) |

IPTW, inverse probability of treatment weighting; IQR, interquartile range

^a^ Defined by CDC: <https://www.cdc.gov/covid/hcp/clinical-care/underlying-conditions.html>

^b^ The data set captures only a small subset of all lab tests for COVID-19 as the values have to be captured within one of the contributing outpatient EHR sources.

Supplementary Table 5. Unadjusted and adjusted vaccine effectiveness estimates

|  | **Unadjusted VE*, (95% CI)** | | **Adjusted VE**, (95% CI)** | |
| --- | --- | --- | --- | --- |
|  | COVID-19-related hospitalization | Any medically-attended COVID-19 | COVID-19-related hospitalization | Any medically-attended COVID-19 |
| All adults (≥18 years) | 68.5% (62.9%-73.2%) | 36.5% (33.7%-39.1%) | 60.2% (53.4%-66.0%) | 33.1% (30.2%-35.9%) |
| ≥18 years and high risk^a^ | 65.5% (59.1%-70.8%) | 36.1% (32.9%-39.1%) | 58.7% (51.3%-65.0%) | 34.5% (31.2%-37.6%) |
| ≥50 years | 69.0% (63.4%-73.8%) | 38.4% (35.5%-41.1%) | 61.1% (54.3%-66.9%) | 35.3% (32.2%-38.2%) |
| ≥65 years | 68.8% (62.9%-73.8%) | 42.6% (39.4%-45.5%) | 60.5% (53.3%-66.6%) | 38.7% (35.4%-41.9%) |

^a^ Defined by CDC: <https://www.cdc.gov/covid/hcp/clinical-care/underlying-conditions.html>

* Pre weighing **post weighing
